# Optimization of Flavonoids Extraction Process in *Panax notoginseng* Stem Leaf and a Study of Antioxidant Activity and Its Effects on Mouse Melanoma B16 Cells

**DOI:** 10.3390/molecules23092219

**Published:** 2018-09-01

**Authors:** Chun-Yan Dai, Peng-Fei Liu, Pei-Ran Liao, Yuan Qu, Cheng-Xiao Wang, Ye Yang, Xiu-Ming Cui

**Affiliations:** 1 College of Life Science and Technology, Kunming University of Science and Technology, Kunming 650500, China; daichunyankm@126.com (C.-Y.D.); ragnarok928@sina.com (P.-F.L.); westpp@126.com (P.-R.L.); quyuan2001@126.com (Y.Q.); wcx1192002@126.com (C.-X.W.); 2 Yunnan Key Laboratory of Sustainable Utilization of Panax Notoginseng, Kunming 650500, China; 3 Laboratory of Sustainable Utilization of Panax Notoginseng Resources, State Administration of Traditional Chinese Medicine, Kunming 650500, China; 4 University Based Provincial Key Laboratory of Screening and Utilization of Targeted Drugs, Kunming 650500, China

**Keywords:** antioxidant, flavonoids, mouse melanoma B16 cells, *Panax notoginseng*, surfactant

## Abstract

The *Panax notoginseng* (*P. notoginseng*) stem leaf is rich in flavonoids. However, because of a lack of research on the flavonoid extraction process and functional development of *P. notoginseng* stem leaf, these parts are discarded as agricultural wastes. Therefore, in this study, we intend to optimize the extraction process and develop the skin-whitening functions of *P. notoginseng* stem leaf extracts. The extraction process of the stem and leaf of *P. notoginseng* flavonoid (SLPF) is optimized based on the Box–Behnken design (BBD) and the response surface methodology (RSM). The optimum extraction conditions of the SLPF are as follows: the extraction time, the ethanol concentration, the sodium dodecyl sulfate (SDS) content and the liquid material ratio (*v*/*w*, which are 52 min, 48.7%, 1.9%, and 20:1, respectively. Under the optimal extraction conditions, the average total SLPF content is 2.10%. The antioxidant activity and anti-deposition of melanin of mouse B16 cells of *P. notoginseng* stem leaf extracts are studied. The results indicate that the EC_50_ values of reducing activity, 2,2-diphenyl-1-picrylhydrazyl (DPPH) free radical scavenging activities, the superoxide anion removal ability, and the 2,2-azino-bis-3-ethylbenzthiazoline-6-sulphonic acid (ABTS) free radical removal ability are 7.212, 2.893, 2.949, and 0.855 mg/mL, respectively. The extracts IC_50_ values of the tyrosinase and melanin synthesis are 0.045 and 0.046 mg/mL, respectively. Therefore, the optimal processing technology for the SLPF obtained in this study not only increases its utilization rate, but also decreases material costs. The extracts from the *P. notoginseng* stem leaf may be developed as food or beauty products.

## 1. Introduction

*Panax notoginseng* (*P. notoginseng*) (Burk.) F. H. Chen is a perennial plant belonging to the Araliaceae. It has underground roots that are dried for use as medicine, and was included in the Chinese pharmacopoeia. The *P. notoginseng* stem leaf has a similar medicinal value as the root. It is reported in the Pents’ao Kang Mu that the stem leaf is suitable for the treatment of fractures, bruises, and bleeding. Modern studies have also confirmed that the *P. notoginseng* stem leaf has a medicinal value similar to that of the root for the blood system, the cardiovascular system, the nervous system, and the metabolic system [1]. Common products made from the *P. notoginseng* stem leaf include Qiye Shen’an Pian, Jiang Zhi Ling capsules, and Qiye Shen’an capsules.

Although the stem leaf of *P. notoginseng* has been officially certified as food due to their high nutritional and high medicinal values (DBS 53/024-2017) [2], little attention has focused on promoting these plant parts for food. Preliminary statistics have shown that the annual output of the stem leaf of *P. notoginseng* is 1500 tons and only 5% of this amount is utilized [3]. Therefore, encouraging the development of the stem leaf resources of *P. notoginseng* can greatly increase the income of *P. notoginseng* producers and reduce environmental pollution, thus increasing the socio-economic benefits.

The most effective active ingredients extracted from the stem leaf of *P. notoginseng* are the total saponins and flavonoid glycosides, which account for 4–6% and 0.54–2.49%, respectively [4]. Flavonoids are important because of their well-defined pharmacological activities, which include antioxidant, liver-protective, and anti-tumor activities [5]. The most commonly used organic solvents for extracting flavonoids are ethanol and methanol [6]. For example, Uysal et al. [7] successfully extracted flavonoid-containing components from *Cotoneaster integerrimus* using methanol. Wang et al. [8] used 70% ethanol to extract flavonoids from the leaves of acer truncatum. The extraction rate was higher, and the extract was easier to concentrate and dry. It is considered that ethanol extraction is a better extraction method. At present, only Zhang et al. [4,9] extracted SLPF by a microwave alkali water method and a hot dipping method. However, the ultrasonic method can make the extract continue to shock, contribute to the solute diffusion, shorten the extraction time greatly, and improve the extraction rate of total flavonoids and the use of raw materials, which is a relatively new method for flavonoid extraction [6]. Therefore, the efficient extraction of the SLPF and research on the use of the product for cosmetics and health care are of great significance for the further development of this industry.

Surfactants are widely used as an auxiliary in the extraction of active components; they increase the extraction efficiency, shorten the extraction time, and increase the solubility of the active water-insoluble ingredients in water. In addition, the application of surfactants reduces the use of organic solvents and their cost, optimizes the target components during the extraction process, and improves the purity of the active ingredients. Most of the surfactants used for extraction are non-toxic. Sodium dodecyl sulfate (SDS), Triton X-100, Tween-20, Tween-80, and Span-20 are commonly used surfactants that can significantly increase the extraction rate of flavonoids [10,11,12,13].

The response surface methodology (RSM) has been widely used for process optimization, and the Box–Behnken design (BBD) is one statistical model of the response surface design methods. The BBD represents an independent quadratic design that does not contain an embedded factorial or fractional factorial design. Compared with other design methods, the BBD is easy to design and to analyze statistically; therefore, it is widely used in the extraction process optimization of flavonoids [14]. For instance, the microwave-assisted extraction variables were optimized using the RSM for optimal recoveries of total flavonoid content [15]. In addition, some researchers obtained a better technological condition to extract total flavones from Flos Populi [16] and Coriandrum sativum seeds [17] by using a BBD of the RSM.

Previously, the flavonoid extraction methods have been reported [18], but so far, the extraction process of SLPF has not yet been formed. In this study, the ultrasound-assisted extraction process of SLPF was optimized by using the BBD to determine the content of total flavonoids as an index for the first time. The independent variables, i.e., a surfactant type, dosage, liquid material ratio (*v*/*w*, extraction time, and an ethanol concentration, are set as single factors. Therefore, the objectives of the present work were (i) to optimize the ultrasound-assisted extraction process of total flavonoids; (ii) to evaluate the antioxidant activity of the extracts; (iii) to evaluate the effect of the extracts on B16 cell activity; (iv) to explore the feasibility of the application of the SLPF in cosmetics, healthcare products, and the pharmaceutical industry; (v) to provide a reference for the development of *P. notoginseng* stem leaf extract products and reduce the discharge of agricultural wastes into the environment.

## 2. Results and Discussion

### 2.1. Effect of Surfactant Types on Extraction Efficiency of SLPF

**Figure 1:**
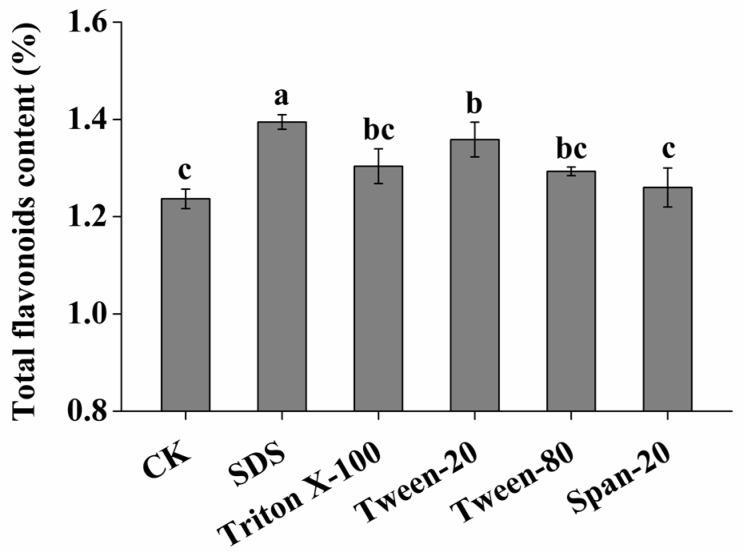
Effects of different types of surfactants on contents of SLPF. Different letters imply significant differences at *p* < 0.05.

In addition to the control group (CK) (water instead of surfactants), different types of surfactants (1.5%) were used in other groups by maintaining liquid material ratio (*v*/*w* of 15:1 and using a 40% ethanol concentration. The total flavonoid content was determined after ultrasonic extraction at 80 °C for 40 min; the results are shown in Figure 1. It can be seen that the surfactants improved the extraction efficiency of the SLPF, and the highest content was extracted by the SDS. The SLPF content using SDS was increased by 12.8% compared to the CK. Therefore, SDS was used in the subsequent experiments.

### 2.2. Single Factor Experiments

**Figure 2:**
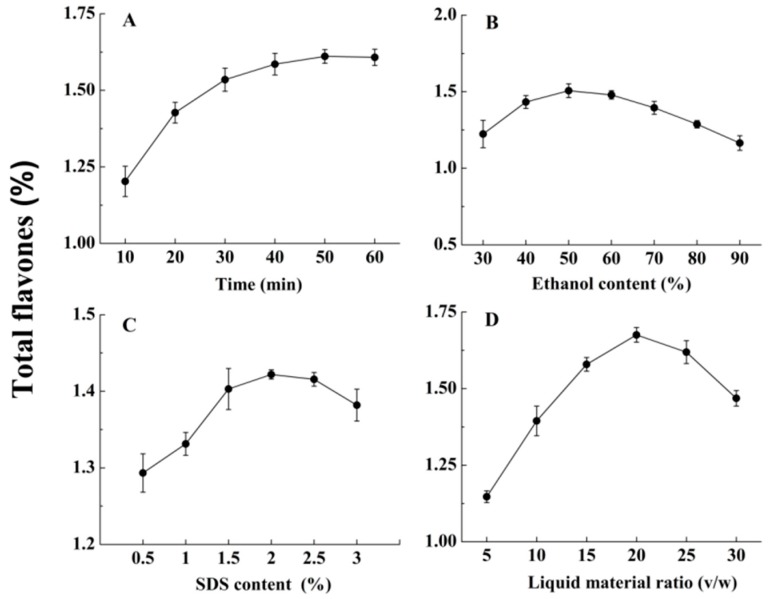
The single factor experiments. Total flavones (%) as a function of (**A**) ultrasound time (min); (**B**) ethanol concentrations (%); (**C**) SDS contents (%) and (**D**) liquid material ratios (*v*/*w*).

The result showed that the extraction efficiency of SLPF increased with the increase in the extraction time; at liquid material ratio (*v*/*w*) of 15:1, the ethanol concentration was 40% and the SDS content was 1.5% (Figure 2A). The yield of the SLPF content decreased when the extraction time was longer than 40 min. The SLPF content increased when the ethanol concentration increased from 30% to 50%, but decreased gradually when the ethanol concentration was higher than 50%. The highest yield of the SLPF content was 23.1% for extraction time of 40 min, liquid material ratio (*v*/*w*) of 15:1, and an SDS content of 1.5% (Figure 2B). When the extraction time was 40 min, the liquid material ratio (*v*/*w*) was 15:1, and the ethanol concentration was 40%, the SLPF content continued to increase with the SDS content from 0.5% to 2%. When the SDS content was increased from 0.5% to 2%, and the SLPF content was increased by 10% (Figure 2C). The total flavonoid content was highest when the liquid material ratio (*v*/*w*) was 20:1, the extraction time was 40 min, the ethanol concentration was 40%, and the SDS content was 1.5% (Figure 2D). The SLPF content was 1.5 times higher than that when liquid material ratio (*v*/*w*) of 5:1 was used, and decreased when the liquid material ratio was greater than 20:1. Therefore, for the subsequent BBD experiments, extraction time ranging from 40 to 60 min, ethanol concentrations ranging from 40% to 60%, SDS contents ranging from 1.5% to 2.5%, and the liquid material ratio (*v*/*w*) ranging from 15:1 to 25:1 were used.

### 2.3. Model Fitting and Optimization of SLPF

#### 2.3.1. Model Fitting

The designed matrix, the results of the analysis of variance, and the adequacy and fitness of the models are shown in Table 1. A multiple regression analysis was conducted using the Design–Expert software. The relationship between the response variable (the content of SLPF) and the independent variables was expressed by the following second-order polynomial equation:

Y = −30.678 + 0.487A + 0.304B + 4.596C + 0.853D − 0.001AB − 0.020AC − 0.005AD + 0.010BC − 0.001BD − 0.076CD − 0.003A^2^ − 0.002B^2^ − 0.683C^2^ − 0.010D^2^ (1)

where Y is the SLPF content; A, B, C, and D represent the extraction time, the ethanol concentration, the SDS content, and the liquid material ratio, respectively.

#### 2.3.2. Analysis of Response Surface

The residual analysis of the response surface optimization model was conducted using a graphical analysis tool. It is important to test the uniformity of the error variance of the model. If the results exhibit a good fit with the predicted values, the fitted curve of the experimental and predicted values is linear. Moreover, it is important to evaluate if the distribution of the residuals is normal when determining the model accuracy. When the residuals have a normal distribution, the fitted curve of the residuals is linear [19]. In addition, if the predictive values of the residuals have a random distribution, the homogeneity of the residual variance is consistent with the optimization requirements [20].

**Figure 3:**
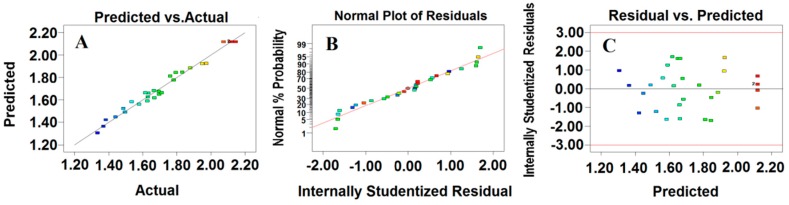
Predicted total flavonoid content versus experimental total flavonoids content (**A**); normal probability plots as a function of residuals (**B**) and plots of the residuals versus the predicted response (**C**).

In this study, the quadratic model had the best fit for the data (*R*^2^ value of 0.9818) (Figure 3A) [21]. In addition, the normal probability plot of the residuals (Figure 3B) and the plot of the residuals versus the predicted response (Figure 3C) were used to validate the adequacy of the model. The data showed that the points in the normal plot formed a straight line (Figure 3B). This indicated that the data exhibited no deviation from normality. In addition, the plot of the residuals versus the predicted response showed a random distribution, which indicated that the model was adequate and did not violate the assumption of independence or constant variance (Figure 3C) [22].

The results showed that a high proportion of the total variance was explained by the quadratic regression model (*R^2^* of 0.982), indicating that the model is suitable for the optimization of the extraction parameters [23]. The *R^2^*_Adj_ of 0.964 was relatively close to the *R^2^*_Pred_ of 0.902, corroborating that the model is significant [14]. The value of the signal-to-noise ratio (*R*_SN_) was 24.553, which was well above 4. The result indicated that this model could be used for the design space. Moreover, the coefficient of variation (CV) was low (2.657%), indicating that the model fitted the experimental data satisfactorily and can be used to predict the outcome within the range of the data. The regression coefficients are listed in Table 2. In the SLPF model, A, B, C, AB, AC, AD, BC, CD, A^2^, B^2^, C^2^, and D^2^ were significantly different (*p* < 0.05), but D and BD were not significantly different (*p* > 0.05).

**Figure 4:**
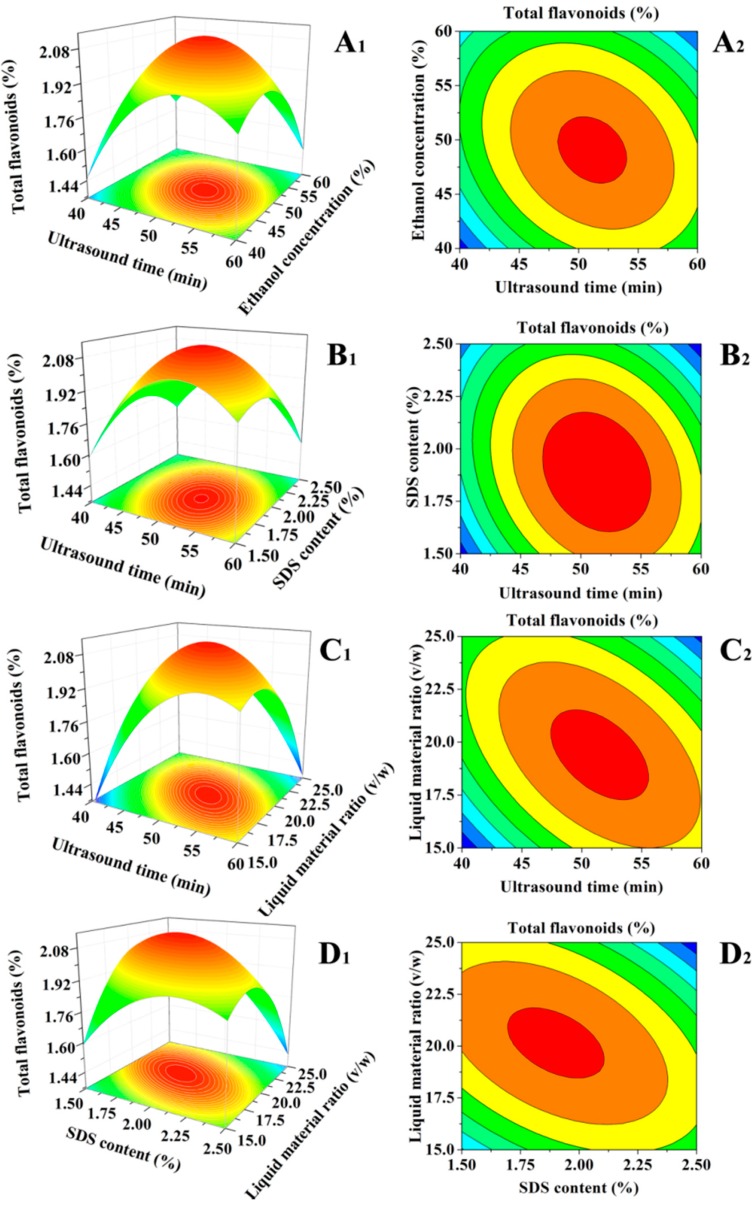
Response surface for the effect of operating parameters on total flavonoid contents; (**A_1_**) ultrasound time vs. ethanol concentration; (**B_1_**) ultrasound time vs. an SDS content; (**C_1_**) ultrasound time vs. liquid material ratio (*v*/*w*); (**D_1_**) an SDS content vs. liquid material ratio (*v*/*w*). (**A_2_**–**D_2_**) are their contour plots, respectively.

A 3D surface graph (Figure 4A_1_–D_1_) of the total flavonoid content and the contour curve (Figure 4A_2_–D_2_) of the two tested variables were generated from the final model to describe the interactions between the independent variables and the optimal process parameters. Each graph was completed on the condition that the other factors were kept at their respective zero levels each time [24]. If the contour plot has a circular shape, the interactions between the corresponding factors are negligible. The elliptical shape of the contour plot indicates that the interactions between the variables contribute to the content of total flavonoids at a significant level [25].

Based on the data shown in Table 2 and Figure 4, in the SLPF model, the ranking of the interaction effect between the independent variables from high to low was CD–AD–AB–AC. The effects of C (the SDS content) and D (the liquid material ratio (*v*/*w*)) on the SLPF content were shown as a 3D plot and an associated contour plot. The elliptical shape of the contour plot illustrated a significant (*p* < 0.0001) correlation between C and D, which contributed to the different SLPF contents (Table 2; Figure 4D_1_,D_2_). The SLPF contents increased (2.13%), when the SDS content was increased from 1.5% to 2.0% and the liquid material ratio (*v*/*w*) was increased from 15:1 to 20:1 (Figure 4D_1_,D_2_). It is important that the extraction process is economical and feasible and the modeling approach facilitates the reduction in the cost and time during future mass production.

#### 2.3.3. Validation of the Model

To validate the proposed model, various reaction conditions were selected within the range of the variables (Table 3). The optimal process parameters for obtaining a high total flavonoid content obtained by Equation (1) were extraction time of 51.84 min, an ethanol concentration of 48.68%, an SDS content of 1.88%, and liquid material ratio (*v*/*w*) of 19.81:1; this resulted in a total flavonoid content of 2.13%. The accuracy of the model was confirmed based on three assays under optimum conditions. The average total flavonoid content was 2.10%, which showed no significant difference from the predicted value. This indicated that the experimental value was very close to the predicted value, which proved that the results were reasonable and reliable; the model of Equation (1) was considered to be satisfactory and accurate for predicting the SLPF content. This approach does not only improve the extraction efficiency, but also increases the utilization rate of the *P. notoginseng* stem leaf.

### 2.4. Antioxidant Activities of the P. notoginseng Stem Leaf Extracts

**Figure 5:**
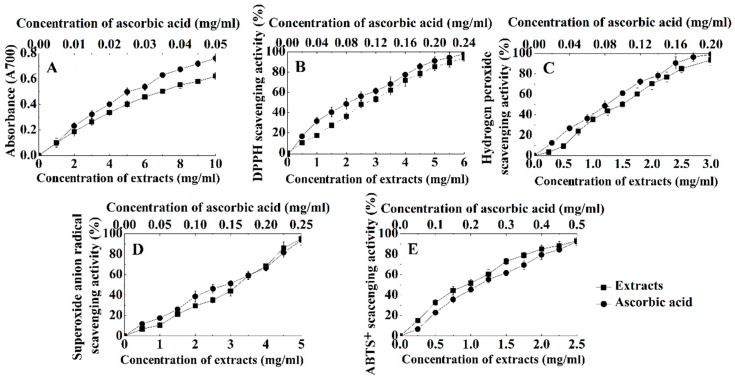
Antioxidant activity of the extracts from *P. notoginseng* stem leaf and ascorbic acid at different concentrations. (**A**–**E**) were the ferric ions reducing activity, the DPPH radical scavenging rate, the hydroxyl radical scavenging rate, the superoxide anion scavenging rate and the ABTS^+^ radical scavenging rate, respectively.

The reduction ability of the *P. notoginseng* stem leaf extracts is positively related to its antioxidant activity; therefore, it can be used as an important evaluation index of antioxidant activity [26]. The reduction abilities of the extracts and of the ascorbic acid increased with the concentration and showed a significant positive correlation with the concentration (Figure 5A). The EC_50_ of the extracts and ascorbic acid were 7.212 and 0.0287 mg/mL, respectively. These results suggested that *P. notoginseng* extracts are an effective electron donor capable of reacting with free radicals to convert them into more stable products.

The DPPH radical is a relatively stable free radical and can accept electrons or hydrogen atoms to form stable molecules. Hydroxyl radicals possess the highest oxidation ability and can damage biological molecules. Therefore, DPPH and hydroxyl radicals have been widely used as an important evaluation index of antioxidant activity [27]. The DPPH radical scavenging activities increased with the concentration of the extracts and ascorbic acid, and they all showed a significant positive correlation with the concentration. The EC_50_ values of the extracts and ascorbic acid were 2.893 and 0.0938 mg/mL, respectively (Figure 5B). The hydroxyl radical scavenging activities had a similar trend as the DPPH radical for the treatments of the extracts or the ascorbic acid, and the EC_50_ values of the extracts and ascorbic acid were 1.514 and 0.0902 mg/mL, respectively (Figure 5C). These results indicated that the *P. notoginseng* extracts have a high ability to remove DPPH or hydroxyl radicals.

Superoxide anions can lead to the occurrence of peroxides in the plasma membrane [28]. Therefore, the ability to remove superoxide anions is extremely important for an antioxidant. In this study, the superoxide anion scavenging rate increased with the concentration of the extracts and ascorbic acid, and the removal rate was also correlated with the concentration (Figure 5D). The EC_50_ values of the extracts and ascorbic acid were 2.949 and 0.140 mg/mL, respectively, and the highest removal rates were 94.90% and 93.73%, respectively.

In addition, the antioxidant activity of the extracts is generally reflected in the ability to remove ABTS^+^ free radicals [29]. The scavenging effect of the extracts and ascorbic acid on the ABTS^+^ free radicals increased with the concentration (Figure 5E), and there was a positive correlation between the scavenging rate and the concentration of the extracts or the ascorbic acid. The EC_50_ values of the *P. notoginseng* extracts and the ascorbic acid were 0.855 and 0.205 mg/mL, respectively.

Many studies have found that flavonoids possess good antioxidant properties. The results of Farombi et al. [30] showed that an extract of *Garcinia kola* seeds exhibited a 57% scavenging effect on superoxide at a concentration of 1 mg/mL, an 85% scavenging effect on hydrogen peroxide at a concentration of 1.5 mg/mL, and an 89% scavenging effect on DPPH at a concentration of 2 mg/mL. Srisawat et al. [31] demonstrated the superoxide anion radical scavenging activities of rice extracts EC_50_ values in the range of 0.6–5 mg/mL, which was similar to the results of this study. Overall, this research also indicated that the *P. notoginseng* stem leaf extracts possess a strong antioxidant activity.

### 2.5. Effect of Extracts on B16 Cell Activity

**Figure 6:**
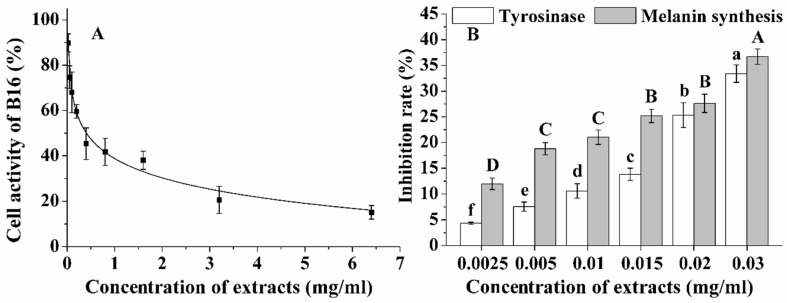
The cell activity (**A**) and the inhibition rate of tyrosinase and melanin synthesis (**B**) in B16 cells. Capital letters indicate differences in the inhibition rate of melanin synthesis under different treatment; lowercase letters indicate differences in the inhibition rate of tyrosinase under different treatment (*p* < 0.05).

The safety of the *P. notoginseng* stem leaf extracts is extremely important in the investigation of cell viability, cellular tyrosinase activity and melanin levels in B16 melanoma cells. The results showed that the cell viability was about 85% at a concentration of 0.032 mg/mL and that the IC_50_ value of the cell viability was 0.405 mg/mL (Figure 6A). The *P. notoginseng* stem leaf extracts were not especially cytotoxic to B16 cells. Therefore, extract concentrations lower than 0.032 mg/mL were used to perform the subsequent experiments.

Tyrosinase is a rate-limiting, regulatory melanogenic enzyme that is involved in the melanin synthesis pathway [32]. The results indicated that the extracts significantly reduced the tyrosinase activity and melanin production in the murine cells in a dose-dependent manner (Figure 6B). There were positive correlations between the extracted content and the inhibition ratio of the tyrosinase or the melanin synthesis (*R^2^* = 0.9162 and *R^2^* = 0.9118). The IC_50_ values of the inhibition ratio of the tyrosinase and melanin synthesis were 0.045 mg/mL and 0.046 mg/mL, respectively (Figure 6B).

Melanin plays a decisive role in skin color. The formation of melanin is regulated by tyrosinase, which is a major rate-limiting enzyme that is regulated by free radicals. Detailed studies have shown that flavonoids are rather potent inhibitors, and can directly inhibit tyrosinase and act on the distal part of the melanogenesis oxidative pathway [33]. Our results indicated that the *P. notoginseng* stem leaf extracts exhibited good performance in reducing the tyrosinase activity and melanin production. It is evident that *P. notoginseng* stem leaf extracts may have great prospects as health food and a cosmetic product because of its excellent performance in anti-pigment deposition.

## 3. Materials and Methods

### 3.1. Plant Materials

Fresh stem leaf of *P. notoginseng* was bought from the international trading center of *P. notoginseng*, Wenshang, Yunnan province, in October 2017, and then washed in distilled water, and surface water was removed. The fresh stem leaf of *P. notoginseng* was dried to a constant weight in 60 °C, and were then powdered for further use.

### 3.2. Chemicals and Reagents

Na_2_HPO_4_, NaH_2_PO_4_, TCA (trichloroacetic acid), FeCl_3_, FeSO_4_, ascorbic acid, pyrogallol, H_2_O_2_ were obtained from Sinopharm Chemical Reagent Co., Ltd. (Shanghai, China) DPPH (2,2-diphenyl-1-picrylhydrazyl), ABTS, α-MSH, L-DOPA and MTT were purchased from Sigma Chemical Co. (St. Louis, MO, USA), PBS (Biological Industries, Beit-Haemek, Israel), Dulbecco’s Modifed Eagle Medium (DMEM) culture and fetal bovine serum (FBS; Gibco, NY, USA), and trypsin were obtained from Biological Industries, Israel. DMSO and Triton X-100 were obtained from MP Biomedicals and Amresco in USA, respectively. All the other reagents were of analytical grade. Ultrapure water was obtained from the Millipore system (Bedford, MA, USA).

### 3.3. Optimization of Extraction Condition Selection

Ultrasound-assisted extraction of total flavonoids from the *P. notoginseng* stem leaf was optimized by the RSM. The content of SLPF was set as the index to judge the extraction condition. According to the extracting technology, the surfactants, the extraction time, the ethanol concentration, the SDS content and the liquid material ratio are key factors. The highest values in the experiments were the optimal central values for the SLPF content. Then, they were used to study the best extraction process technology of SLPF by the method of RSM. Single factor treatment was as follows:

The *P. notoginseng* stem leaf was (1) extracted using different surfactants (Table 4), ultrasound extraction for 40 min by maintaining the liquid material ratio (15:1, *v*/*w*), and 40% ethanol concentration; (2) extracted by ultrasound for 10, 20, 30, 40, 50 and 60 min when the liquid material ratio was 15:1 (*v*/*w*), the concentration of ethanol was 40%, and the content of SDS was 1.5%; (3) extracted by 30, 40, 50, 60, 70, 80 and 90% ethanol for 40 min when the liquid material ratio was at 15:1 (*v*/*w*), and the SDS content was 1.5%; (4) extracted by 0.5, 1, 1.5, 2, 2.5 and 3% SDS for 40 min when the liquid material ratio (*v*/*w*) was 15:1 (*v*/*w*), and the content of ethanol was 40%; (5) extracted with different liquid material ratios (5:1, 10:1, 15:1, 20:1, 25:1 and 30:1; *v*/*w*) for 40 min by maintaining the concentration of ethanol was 40%, and the content of SDS was 1.5%. Then, the content of SLPF in extracts was determined.

### 3.4. Experimental Design of RSM

Based on the results analysis of single factor experiments, the SLPF extraction condition from *P. notoginseng* stem leaf was developed and optimized using three-levels, four-factor BBD, combined with RSM (Design–Expert Software, trial version 8.1.0; Stat-Ease Inc., Minneapolis, MN, USA). Each independent variable was coded at three levels between −1 and +1, where the time (A); the ethanol concentration (B); the SDS content (C); and the liquid material ratio (D) were changed in the ranges shown in Table 5. The SLPF content was the response variable. For statistical calculations, a second-order quadratic equation was used as follows: [34].

(2) $$Y=\beta_{0}+\overset{4}{\underset{i=1}{\sum }}\beta_{i}X_{i}+\overset{4}{\underset{i=1}{\sum }}\beta_{ii}X_{i}^{2}+\overset{3}{\underset{i=1}{\sum }}.\overset{4}{\underset{j=i+1}{\sum }}\beta_{ij}X_{i}X_{j}+\overset{4}{\underset{i=1}{\sum }}\beta_{iii}X_{i}^{3}$$

where *Y* is the predicted response, *β_0_* is an intercept coefficient, *β_i_* is the linear terms, *β_ii_* is the squared terms, *β_ij_* is the interaction terms, and *X_i_* and *X_j_* represent the coded levels of independent variables.

### 3.5. Total Flavonoid Assay

The content of total flavonoids was determined based on an established method with some modifications, and rutin was selected as the reference standard [35]. The dried rutin was weighed 10 mg accurately, dissolved with 20 mL ethanol and transferred into a 100 mL volumetric flask by adding water to scale. A series of standard solutions of rutin in the range of 0.0–6.0 mL were precisely transferred to 25 mL volumetric flasks. Then, 1 mL of 5% NaNO_2_ was added; after 6 min, 1.0 mL of 10% Al(NO_3_)_3_ was added and the solution was kept for 6 min at room temperature; 10 mL of 4% NaOH was added to the volumetric flasks. This solution was then diluted with water solution to 25 mL and kept for 15 min at room temperature. The absorbance against a blank sample without rutin (A) was measured at the wavelength of 510 nm using a UV-2600 spectrophotometer (Shanghai Yuanxi Instruments Co. Ltd., Shanghai, China) to obtain the linear response (A = 4.6971B − 0.0072, *R*^2^ = 0.9992). B was the total flavonoids content (mg) at the wavelength of 510 nm. An aliquot of 0.1 mL of the samples were diluted with methanol to 50 mL and was then determine the concentration of total flavonoids at the spectrophotometer. The total flavonoid content was calculated according to the following equation:

(3) $$y=\frac{m\times V}{M}\times 100\%$$

where *m* is total flavonoid content, *V* is the volume of extraction solution and M is the quality of extracts.

### 3.6. In Vitro Antioxidant Activity Assays

We researched the antioxidant activities using different concentrations of extracts. L-ascorbic acid was used to prepare a standard solution and compared with the samples. All the samples were conducted for 5 times.

#### 3.6.1. Reducing Power Assay

The ferrous-ion-reducing ability of extracts assay was carried out according to Park’s [36] method with slight modifications. Briefly, different concentrations of extracts were mixed with a sodium phosphate buffer (2.5 mL, 0.2 mg/mL, pH 6.6) and potassium ferricyanide (2.5 mL, 1%, *w*/*v*) in the test tube, followed by incubation in a water bath at 50 °C for 20 min. Then, trichloroacetic acid (2.5 mL, 10%, *w*/*v*) was added to the mixture to end the reaction. In addition, the solution was centrifuged at 3000 rpm for 10 min. Finally, the supernatant (2.5 mL) was mixed with the deionized water and the FeCl_3_ (0.5 mL, 0.1%, *w*/*v*). The reaction mixture was left at 25 °C for 10 min in the dark and determined the absorbance at 700 nm.

#### 3.6.2. DPPH Radical Scavenging Capacity

The DPPH radical scavenging capacity was determined by Qu’s method with some slight modifications [37]. Different concentrations of extracts (1 mL) were mixed with 1 mL of a 0.002 mg/mL DPPH solution. After the mixtures were incubated at room temperature in the dark for 0.5 h, the discolorations were measured at 517 nm. The scavenging percentage was calculated by the following equation:

(4) $$\mathrm{DPPH}\;\mathrm{radical}\;\mathrm{scavenging}\;\mathrm{activity}\;\left(\%\right)=\left(1-\frac{A_{s}}{A_{0}}\right)\times 100\%$$

where *A_s_* means the absorbance of a sample, and *A*_0_ means the blank control solution without a sample.

#### 3.6.3. Detection of ABTS^+^ Scavenging Assay

ABTS scavenging activity of extracts was quantified by Wang’s method with some modifications [38]. ABTS^+^ (0.007 mg/mL) was dissolved into a potassium persulphate solution (0.00245 mg/mL). The reaction mixture was left at 25 °C for 12 h in the dark before use. The mixture was diluted with 75% of ethanol to adjust its absorbance to 0.70 ± 0.02 at 734 nm. Extracts with different concentrations (0.1 mL) were added into 3.9 mL of this ABTS^+^ solution, and were then incubated at room temperature for 10 min. The absorbance was measured at 734 nm. Ascorbic acid was used as the reference compound for measuring the ABTS^+^ scavenging activity. The scavenging effect of ABTS^+^ was defined as:

(5) $$\mathrm{Scavenging}\;\mathrm{ability}\;\left(\%\right)=\left(1-\frac{A_{s}}{A_{0}}\right)\times 100\%$$

where *A_s_* means the absorbance of a sample and *A*_0_ means the blank control solution without a sample.

#### 3.6.4. Assay of Hydroxyl Radical Scavenging Activity

The radical scavenging capability was measured according to a previously described method with minor modifications [39]. Two milliliter of FeSO_4_ (0.006 mg/mL) and 2.0 mL different concentrations of the extracts were mixed. Then, 2.0 mL H_2_O_2_ (0.006 mg/mL) was added in the mixture to start the reaction. After shaken, the mixture was incubated at 37 °C for 30 min. Then, 2.0 mL of salicylic acid (0.006 mg/mL) was added, mixed and incubated at 37 °C for 10 min. The absorbance was measured at 510 nm. Ascorbic acid was used for comparison. The capability of hydroxyl radical scavenging activity was calculated by the following equation:

(6) $$\mathrm{Scavenging}\;\mathrm{ability}\;\left(\%\right)=\left(1-\frac{A_{1}-A_{2}}{A_{0}}\right)\times 100\%$$

where *A*_1_ and *A*_2_ are the absorbance of a sample (with and without hydrogen peroxide, respectively), and *A*_0_ was the absorbance of a background solution.

#### 3.6.5. Detection of Superoxide Anion Scavenging Activity

The superoxide anion radical scavenging activity of extracts was assayed following the methods described by İlhami Gülçin [40]. The beginning of reaction was to put 2 mmol NADH (20 μL) into the mixture (180 μL), which contained different concentrations of extracts (10 μL), and 1 mmol NBT (20 μL), 0.1 mmol PMS (20 μL), a 250 mmol potassium phosphate buffer (pH 7.4, 40 μL) and water (90 μL), and was then incubated at 25 °C for 20 min. The absorbance of the resulting solution was measured at 570 nm, and the capability of superoxide anion scavenging activity was calculated by the following equation:

(7) $$\mathrm{Superoxide}\;\mathrm{anion}\;\mathrm{radical}\;\mathrm{scavenging}\;\mathrm{activity}\;\left(\%\right)=\left(1-\frac{A_{s}}{A_{c}}\right)\times 100\%$$

where *A*_s_ and *A*_c_ are the absorbance of a sample and a control solution, respectively.

### 3.7. Cell Experiment

#### 3.7.1. Cell Culture

Mouse melanoma cell lines, B16 (obtained from Kunming Institute of Zoology (CAS), Kunming, Yunnan, China) were cultured in DMEM supplemented with 10% fetal bovine serum and 1% penicillin/streptomycin. The cells were incubated in a humidified atmosphere of 5% CO_2_ at 37 °C.

Effects of the extractive on viability of B16 cells were determined using the MTT assay [41]. Cells were seeded into a 6-well plate (10^5^ cells/well) and incubated with samples at different concentrations. Cells were labelled with the MTT solution at 37 °C for 3 h after 48 h of incubation. The violet formazan precipitates were dissolved in DMSO, and then the absorbance of each well, using a microplate reader with a 630 nm reference, was determined at 590 nm.

#### 3.7.2. α-MSH Treatment

B16 cells were cultured for 24 h at a density of 10^5^ cells/mL in 6-well plates, which contained 10% fetal bovine serum and 1% penicillin/streptomycin at 37 °C in a humidified atmosphere with 5% CO_2_. The medium was substituted by a fresh supplement (α-MSH) and different concentrations of extracts and incubated for 48 h. All experiments reported in next sections were performed with these stimulate cells by using the above procedure.

#### 3.7.3. Intracellular Tyrosinase Activity

Tyrosinase enzyme activity was estimated by measuring the rate of L-DOPA oxidation [42]. α-MSH-stimulated cells were treated in the absence or the presence of samples (0.0025–0.03 mg/mL) for 48 h. The cells were then solubilized in a phosphate buffer (0.1 M; pH 6.8) containing Triton X-100 (0.1%). Cellular lysates were centrifuged at 12,000 rpm at 4 °C for 20 min. The cellular extract was incubated with L-DOPA (1.25 mM), and the absorbance was followed spectrophotometrically at 475 nm until the reaction finished.

#### 3.7.4. Melanin Content Assay

The α-MSH-stimulated cells were cultured at a density of 10^5^ cells/mL in 6-well plates for 48 h in the absence or the presence of samples (0.0025–0.03 mg/mL). The cells were washed twice in PBS and dissolved in NaOH (1 mL, 10%) at 100 °C for 1 h. The cell lysates were centrifuged at 1000 g for 10 min, and then the absorbance value of the supernatant was measured at 405 nm using a standard curve of synthetic melanin. The inhibition rate of melanin synthesis of the cells was estimated as a percentage of the control culture [43].

### 3.8. Statistical Analysis

All experiments data were processed with Microsoft Excel software 2010 and the curves were fitted with Origin 8.0. Statistical analysis was carried out by using SPSS 19.0.

## 4. Conclusions

Ultrasound-assisted extraction enhanced the extraction efficiency of total flavonoids. The optimum extraction conditions of SLPF were: ultrasound-assisted extraction time of 52 min, an ethanol concentration of 48.7%, a surfactant SDS content of 1.9%, and liquid material ratio (*v*/*w* of 20:1; this resulted in a 2.10% SLPF content. It was recommended that producers adjusted these data for the extraction of SLPF based on the specific conditions to achieve large-scale and efficient production. The *P. notoginseng* stem leaf extracts exhibited a strong antioxidant activity and induced a decrease in the melanin synthesis by inhibiting the tyrosinase activity. Our results promote the use of *P. notoginseng* stem leaf extracts for cosmetic preparations and food uias a skin whitening agent.

## Figures and Tables

**Table 1:** Experimental design and results for response surface analysis.

Std. Order	Run Order	Variable				Total Flavonoids Y (%)	
		A	B	C	D	Observed	Predicted
25	1	50 (0)	50 (0)	2 (0)	20:1 (0)	2.127	2.117
8	2	50 (0)	50 (0)	2.5 (1)	25:1 (1)	1.384	1.422
11	3	40 (−1)	50 (0)	2 (0)	25:1 (1)	1.781	1.776
22	4	50 (0)	60 (1)	2 (0)	15:1 (−1)	1.615	1.662
12	5	60 (1)	50 (0)	2 (0)	25:1 (1)	1.369	1.364
20	6	60 (1)	50 (0)	2.5 (1)	20:1 (0)	1.579	1.562
1	7	40 (−1)	40 (−1)	2 (0)	20:1 (0)	1.441	1.448
6	8	50 (0)	50 (0)	2.5 (1)	15:1 (−1)	1.794	1.844
10	9	60 (1)	50 (0)	2 (0)	15:1 (−1)	1.949	1.922
17	10	40 (−1)	50 (0)	1.5 (−1)	20:1 (0)	1.535	1.584
9	11	40 (−1)	50 (0)	2 (0)	15:1 (−1)	1.335	1.306
21	12	50 (0)	40 (−1)	2 (0)	15:1 (−1)	1.630	1.626
27	13	50 (0)	50 (0)	2 (0)	20:1 (0)	2.127	2.117
2	14	60 (1)	40 (−1)	2 (0)	20:1 (0)	1.762	1.811
16	15	50 (0)	60 (1)	2.5 (1)	20:1 (0)	1.711	1.663
7	16	50 (0)	50 (0)	1.5 (−1)	25:1 (1)	1.973	1.924
23	17	50 (0)	40 (−1)	2 (0)	25:1 (1)	1.694	1.678
28	18	50 (0)	50 (0)	2 (0)	20:1 (0)	2.145	2.117
14	19	50 (0)	60 (1)	1.5 (−1)	20:1 (0)	1.666	1.683
29	20	50 (0)	50 (0)	2 (0)	20:1 (0)	2.074	2.117
15	21	50 (0)	40 (−1)	2.5 (1)	20:1 (0)	1.669	1.618
4	22	60 (1)	60 (1)	2 (0)	20:1 (0)	1.496	1.490
13	23	50 (0)	40 (−1)	1.5 (−1)	20:1 (0)	1.832	1.846
18	24	60 (1)	50 (0)	1.5 (−1)	20:1 (0)	1.879	1.885
5	25	50 (0)	50 (0)	1.5 (−1)	15:1 (−1)	1.627	1.590
19	26	40 (−1)	50 (0)	2.5 (1)	20:1 (0)	1.634	1.659
26	27	50 (0)	50 (0)	2 (0)	20:1 (0)	2.114	2.117
24	28	50 (0)	60 (1)	2 (0)	25:1 (1)	1.486	1.522
3	29	40 (−1)	60 (1)	2 (0)	20:1 (0)	1.697	1.649

**Table 2:** The results of analysis on variance (ANOVA) for the effects of variables.

Source	Sum of Squares	DF	Mean Square	F Value	*p* Value Prob > F	Significance
Model	1.589	14	0.114	53.850	<0.0001	***
A	0.031	1	0.031	14.757	0.0018	**
B	0.011	1	0.011	5.026	0.0417	*
C	0.046	1	0.046	21.826	0.0004	***
D	0.006	1	0.006	2.750	0.1195	
AB	0.068	1	0.068	32.313	<0.0001	***
AC	0.040	1	0.040	18.891	0.0007	***
AD	0.263	1	0.263	124.984	<0.0001	***
BC	0.011	1	0.011	5.165	0.0393	*
BD	0.009	1	0.009	4.360	0.0555	
CD	0.143	1	0.143	67.750	<0.0001	***
A^2^	0.487	1	0.487	230.873	<0.0001	***
B^2^	0.386	1	0.386	182.935	<0.0001	***
C^2^	0.189	1	0.189	89.704	<0.0001	***
D^2^	0.409	1	0.409	194.231	<0.0001	***
Residual	0.030	14	0.002			
Lack of Fit	0.027	10	0.003	3.858	0.1025	
Pure Error	0.003	4	0.001			
Cor Total	1.619	28				
						
Std. Dev.	0.046		*R* ^2^	0.982		
Mean	1.728		*R* ^2^_Adj_	0.964		
C.V. (%)	2.657		*R* ^2^_Pred_	0.902		
PRESS	0.158		*R*_SN_	24.553		

DF = degree of freedom. PRESS = predicted residual sum of squares. Cor Total = correlation total. *R^2^*_Adj_ = adjusted *R*^2^. Std. Dev. = standard deviation. *R^2^*_Pred_ = predicted *R*^2^. C.V. (%) = coefficient of variation percent. *R*_SN_ = signal-to-noise ratio.

**Table 3:** The optimization criteria of the maximum total flavonoid content and the results of model validation.

Name	Goal	Lower Limit	Upper Limit	Lower Weight	Upper Weight
A: Ultrasound time (min)	is in range	40	60	1	1
B: Ethanol concentration (%)	is in range	40	60	1	1
C: SDS content (%)	is in range	1.5	2.5	1	1
D: Liquid material ratio (*v*/*w*)	is in range	15	25	1	1
Total flavonoids content (%)	maximize	1.33502	100	1	1
Category	Ultrasound time (min)	Ethanol concentration (%)	SDS content (%)	Liquid material ratio (*v*/*w*)	Total flavonoids content (%)
Predicted value	51.84	48.68	1.88	19.81:1	2.13 ± 0.008a
Experimental value	52	48.7	1.9	20:1	2.10 ± 0.017a

Note: The little letters “a” means that there is no significant difference between the predicted value and the experimental value of the total flavonoid content at *p* < 0.05.

**Table 4:** Surfactant types.

Surfactant	SDS	Triton X-100	Tween-20	Tween-80	Span-20
HLB	40	14.6	16.7	15	8.6
Types	Anionic	Nonionic	Nonionic	Nonionic	Nonionic

**Table 5:** Levels and codes of independent variable used for response surface analysis.

Factors	Codes	Levels		
		−1	0	1
Ultrasound time (min)	A	40	50	60
Ethanol concentration (%)	B	40	50	60
SDS content (%)	C	1.5	2	2.5
Liquid material ratio (*v*/*w*)	D	15:1	20:1	25:1

## Abbreviations

ABTS	2,2-azino-bis-3-ethylbenzthiazoline-6-sulphonic acid
BBD	Box-Behnken design
DPPH	2,2-diphenyl-1-picrylhydrazyl
RSM	Response surface methodology
SLPF	stem and leaf of *P. notoginseng* flavonoid
